# Two-Stage, *In Silico* Deconvolution of the Lymphocyte Compartment of the Peripheral Whole Blood Transcriptome in the Context of Acute Kidney Allograft Rejection

**DOI:** 10.1371/journal.pone.0095224

**Published:** 2014-04-14

**Authors:** Casey P. Shannon, Robert Balshaw, Raymond T. Ng, Janet E. Wilson-McManus, Paul Keown, Robert McMaster, Bruce M. McManus, David Landsberg, Nicole M. Isbel, Greg Knoll, Scott J. Tebbutt

**Affiliations:** 1 PROOF Centre of Excellence, Vancouver, BC, Canada; 2 Department of Statistics, University of British Columbia, Vancouver, BC, Canada; 3 Department of Computer Science, University of British Columbia, Vancouver, BC, Canada; 4 Department of Medicine, Division of Nephrology, University of British Columbia, Vancouver, BC, Canada; 5 Department of Medical Genetics, University of British Columbia, Vancouver, BC, Canada; 6 Department of Pathology and Laboratory Medicine, University of British Columbia, Vancouver, BC, Canada; 7 Department of Medicine, Division of Respiratory Medicine, University of British Columbia, Vancouver, BC, Canada; 8 UBC James Hogg Centre for Heart Lung Innovations, Vancouver, BC, Canada; 9 Division of Nephrology, St. Paul's Hospital, and University of British Columbia, Vancouver, BC, Canada; 10 Department of Nephrology, Princess Alexandra Hospital, and University of Queensland, Brisbane, Australia; 11 Ottawa Hospital Research Institute, Ottawa, On, Canada; Centro Cardiologico Monzino IRCCS, Italy

## Abstract

Acute rejection is a major complication of solid organ transplantation that prevents the long-term assimilation of the allograft. Various populations of lymphocytes are principal mediators of this process, infiltrating graft tissues and driving cell-mediated cytotoxicity. Understanding the lymphocyte-specific biology associated with rejection is therefore critical. Measuring genome-wide changes in transcript abundance in peripheral whole blood cells can deliver a comprehensive view of the status of the immune system. The heterogeneous nature of the tissue significantly affects the sensitivity and interpretability of traditional analyses, however. Experimental separation of cell types is an obvious solution, but is often impractical and, more worrying, may affect expression, leading to spurious results. Statistical deconvolution of the cell type-specific signal is an attractive alternative, but existing approaches still present some challenges, particularly in a clinical research setting. Obtaining time-matched sample composition to biologically interesting, phenotypically homogeneous cell sub-populations is costly and adds significant complexity to study design. We used a two-stage, *in silico* deconvolution approach that first predicts sample composition to biologically meaningful and homogeneous leukocyte sub-populations, and then performs cell type-specific differential expression analysis in these same sub-populations, from peripheral whole blood expression data. We applied this approach to a peripheral whole blood expression study of kidney allograft rejection. The patterns of differential composition uncovered are consistent with previous studies carried out using flow cytometry and provide a relevant biological context when interpreting cell type-specific differential expression results. We identified cell type-specific differential expression in a variety of leukocyte sub-populations at the time of rejection. The tissue-specificity of these differentially expressed probe-set lists is consistent with the originating tissue and their functional enrichment consistent with allograft rejection. Finally, we demonstrate that the strategy described here can be used to derive useful hypotheses by validating a cell type-specific ratio in an independent cohort using the nanoString nCounter assay.

## Introduction

Acute rejection is a major complication of solid organ transplantation that prevents the long-term assimilation of the allograft. It is caused by an immune response, with both innate and adaptive components, mounted by the host against alloantigen in the donor tissue. Various lymphocyte sub-populations are known to be principal mediators of this immune response, infiltrating graft tissues and driving cell-mediated cytotoxicity [1], [2]. Understanding the immune response, and lymphocyte-specific biology, associated with rejection is critical if we are to prevent irreversible damage to the graft and may lead to the development of more targeted and successful tolerance strategies [3].

Measuring genome-wide changes in transcript abundance in circulating blood cells (hereafter peripheral whole blood gene expression) can deliver a comprehensive view of the status of the immune system and has been useful in studying the pathobiology of many diseases, including kidney allograft rejection [4]–[6]. Interpreting the results of gene expression studies carried out in peripheral whole blood cells, however, is complicated by the heterogeneous nature of this tissue. Traditional microarray analysis methods do not take into account sample cell type composition. When considering the results of such analyses, we cannot distinguish between variations in gene expression resulting from actual changes in transcript abundance within one or more of the cell types in the sample under study and differences in cell type frequency [7]. In fact, both of these sources of expression variation are significant contributors to the overall variation seen in peripheral whole blood expression data [8]. Sample heterogeneity necessarily affects our ability to detect differential gene expression in peripheral whole blood studies. More importantly, it makes drawing meaningful inference from the data difficult. The problem is not limited to peripheral whole blood [9], and is seldom addressed in a rigorous manner. This is both a problem and a missed opportunity. Both sample composition and cell type-specific gene expression are biologically pertinent. The ability to study changes in the composition of complex tissue samples over time or under various experimental conditions in a very granular manner via genome-wide expression profiling is appealing. In peripheral whole blood, leukocyte populations are already routinely used in monitoring and diagnostics [10]–[12]. On the other hand, the ability to assess cell type-specific gene expression within a heterogeneous sample would allow for a better understanding of the molecular processes involved in health and disease, particularly in less abundant cell type compartments (*e.g.*; eosinophils, Tregs), whose signal might otherwise be drowned out by the more abundant cell type compartments (*e.g.*; neutrophils). An ability to study both of these systems and their interplay all within the same sample would be very useful.

Experimental separation of the component cell types of complex tissue samples is an obvious solution. Following isolation and quantification, one could perform between-group differential expression analysis for each of the cell types in a tissue to assess cell type-specific gene expression changes in the experimental context. However, experimental methods for isolating cell type subsets from complex tissues, such as fluorescence-activated cell sorting (FACS) or enrichment columns, are expensive and the need to process additional gene expression assays for each cell type of interest further exacerbates the problem. Ignoring the additional costs incurred, such isolation methods rely on the availability of unique cell-surface markers and appropriate antibodies that may or may not exist for all cell types of interest [13]. In the case of clinical research, the limitations of clinical laboratories should also be considered. Collecting and preparing peripheral whole blood for gene expression studies is relatively straightforward, but many clinical laboratories may not be equipped to perform FACS/enrichment protocols on site. Translational research should be mindful of the limitations that exist in the clinical laboratory and, if at all possible, simplicity in sample collection and preparation is preferred. Finally, and perhaps most troubling from a scientific point of view, these isolation techniques may alter the gene expression of the cells under study [14], [15].

The cell-composition-dependent signal and cell-function-dependent signal in complex tissue gene expression data may, however, be statistically deconvolved. Lu *et al.* [16] pioneered statistical deconvolution of microarray data to study the proportions of cells in different phases of the cell cycle in cultures of the yeast *S. Cerevisiae*. Other groups subsequently demonstrated that microarray expression deconvolution can reasonably quantify the constituents of peripheral whole blood [17], [18]. Concurrently, Shen-Orr *et al.* demonstrated that cell-specific gene expression may be inferred from peripheral whole blood expression data using total leukocyte differentials (cell-specific significance analysis of microarrays [csSAM]) [7]. In each of these cases the measured transcript abundance in peripheral whole blood for each gene, in each sample, is modeled as a linear combination of the transcript abundance of that gene in each of the cells comprising that sample. Provided we can estimate one of these two unknown quantities, multiple linear regression can provide an approximate solution for the other [7], [16]–[18]. A number of approaches have been developed more recently [19], [20], including applications to next generation sequencing [21].

For simplicity, inferring cell type-specific expression from the peripheral whole blood sample expression data can be referred to as the forward case of deconvolution, while inferring composition from the peripheral whole blood sample expression data can be referred to as the reverse case of deconvolution.

These approaches are complementary. While total leukocyte differentials have been proposed as a readily available source of composition information to enable forward deconvolution approaches in a clinical setting, they offer insufficient granularity, most obviously within the lymphocyte (lymphoid) compartment. Flow cytometry can provide much higher granularity, but at higher cost and increased complexity. The use of reverse deconvolution addresses these issues directly by providing more granular composition data without incurring any additional costs or requiring additional data collection. More generally, the use of whole genome expression data to infer cell type composition could theoretically allow for quantification and study of otherwise non-trivially isolatable cell types *in situ*. Finally, we have previously hypothesized that the sensitivity of forward deconvolution may be poor when phenotypically heterogeneous compartments are used in the model (*e.g.*; lymphocyte compartment when using total leukocyte differentials) [22], [23]. In this case, the ability to infer the composition of a complex tissue to an arbitrarily granular level, thus ensuring phenotypically homogeneous component cell types, could result in additional discovery when performing forward deconvolution. This is the main motivation for a combinatorial deconvolution approach.

We describe below the implementation of a two-stage, *in silico* deconvolution strategy and its application to the study of the lymphocyte compartment of peripheral whole blood during acute kidney allograft rejection to highlight its utility. First, we detail the construction of a suitable basis matrix: an estimate of the cell type-specific expression profiles of the various components of peripheral whole blood that allows us to infer the fractions of these components in each sample from the observed expression in peripheral whole blood (reverse deconvolution), and we establish its performance in three separate cohorts of transplant recipients. Next, we demonstrate that including lymphocyte sub-populations may yield additional discovery when performing cell type-specific differential expression analysis in peripheral whole blood and provide a more relevant biological context for this discovery. We establish the plausibility of the cell type-specific probe-sets identified by this approach. Finally, we apply this two-stage, *in silico* deconvolution approach to a timecourse study of acute kidney allograft rejection, including samples and time points for which no independent composition data was available, to highlight its utility when attempting to derive value from existing clinical samples.

## Methods

### Ethics Statement

This prospective observational study was conducted at 4 renal transplant centres (including St. Paul's Hospital and Vancouver General Hospital (Vancouver, BC, Canada) between January 2005 and September 2009, and expanding to include The Ottawa Hospital (Ottawa, ON, Canada) and Princess Alexandra Hospital (Brisbane, Queensland,Australia) between September 2009 and May 2012) and was approved by the UBC Providence Health Care Research Ethics Board (UBC-PHC REB; St. Paul's Hospital, Vancouver, BC), UBC Clinical Research Ethics Board (UBC CREB; Vancouver General Hospital, Vancouver, BC), Ottawa Hospital Research Ethics Board (Ottawa Hospital, Ottawa, ON), and Metro South Health District Human Research Ethics Committee (Princess Alexandra Hospital, Brisbane, Australia), respectively. All eligible patients undergoing a kidney transplant were invited to participate in the study. Recipients eligible for the study were >18 years of age and able to provide informed consent. Recipients who were under 18 years of age, received multiple, different solid organ transplants, HIV positive, or received organs from donors who tested positive for HIV were excluded. All study participants provided informed written consent.

### Kidney Rejection Timecourse Cohort

Recruited patients received a standardized treatment protocol including basiliximab 20 mg i.v. on days 0 and 4, methylprednisolone 125 mg i.v. on the day of transplantation tapering to zero by day 3 post-transplant, tacrolimus 0.075 mg/kg b.i.d. and mycophenolate 1000 mg b.i.d. Tacrolimus concentrations were measured by tandem mass spectrometry, and the dose was adjusted to achieve 12-hour trough levels of 8–12 ng/mL in month 1, 6–9 ng/ml in month 2, and 4–8 ng/ml thereafter. Allograft rejection was diagnosed by normal clinical and laboratory parameters, confirmed by biopsy, and graded according to the Banff 97 working classification of renal allograft pathology [24]. Banff categories 2 and 4 (antibody-mediated or acute/active cellular rejection) were considered significant. Subjects with borderline changes (category 3) are not considered in the current study. There was no patient loss to follow-up during the study. Blood samples were obtained in PAXgene tubes (BD Diagnostics, Franklin Lakes, NJ, USA) immediately prior to transplantation, at 0.5, 1, 2, 3, 4, 8, 12, 26, and 52 weeks post-transplant, and at the time of suspected rejection. Graft tissue was obtained pre-transplant and at the time of all biopsies performed for clinical purposes post-transplant. All samples were stored in a biolibrary until required for analysis.

We employed a case-control design [25] to compare peripheral whole blood composition, peripheral whole blood gene expression and cell-type specific gene expression in subjects with (AR) or without (NR) treatable acute rejection. To ensure precise homogeneous phenotypes, patients were considered eligible for analysis if they were less than 75 years of age; did not have pre-transplant immunosuppression or immunological desensitization; received an AHG-CDC crossmatch negative kidney transplant from a deceased or non-HLA identical living donor; did not receive depleting antibody induction therapy; were able to receive oral immunosuppression, and had no evidence of infection, disease recurrence, and other major co-morbid events. Cases with AR diagnosed during the first 12 months post-transplant were matched as closely as possible for age, sex, degree of sensitization, organ source and date of transplantation with controls (NR) that had no evidence of clinical or acute rejection during the period of follow-up.

This selection process yielded 48 suitable subjects (24 AR, 24 NR, matched) from a primary cohort (kidney transplant recipients from the St. Paul's Hospital and Vancouver General Hospital sites, enrolled between January 2005 and September 2009), described in previous work by our group [5], [6]. Demographics for these subjects are summarized in **Table S1**. Peripheral whole blood gene expression was assayed on Affymetrix U133 Plus 2.0 microarray pre-transplant (baseline), at the time of rejection (rejection) and at the first available time point at least 7 days following rejection (post-rejection). Total leukocyte differentials time-matched to the RNA extraction blood draw (within 24 hours) were available for 41 subjects (18AR, 23 NR), only at the rejection time point.

A secondary cohort of 44 subjects (13 AR, 31 NR) was assembled by applying the same selection process to kidney transplant recipients enrolled between September 2009 and May 2012 across all four sites. Peripheral whole blood gene expression was assayed on the nCounter GX Human Immunology Assay (NanoString Technologies, Seattle, WA, USA) at the time of rejection only. Total leukocyte differentials time-matched to the RNA extraction blood draw were not available for these subjects. This secondary cohort was used to test a cell type-specific hypothesis formulated at the rejection time point in the primary cohort.

### Additional Datasets

The statistical model used to infer the cellular composition of peripheral whole blood samples was constructed, and preliminary validation on it performed, using two aggregate datasets of the expression profiles of leukocyte sub-populations isolated from peripheral whole blood, obtained from the Gene Expression Omnibus (GEO; GSE28490 and GSE28491) website. Two additional groups of patients were used to train and validate the statistical model used to infer the composition of peripheral whole blood samples. The first group, our **training set**, is an aggregate of two previously described cohorts of heart (n = 26) [22], [26] and kidney (GSE20300; n = 24) [7] transplant recipients. Peripheral whole blood gene expression and time-matched total leukocyte differential data was available for all subjects. The second group, an independent cohort of adult kidney transplant recipients (n = 41, a subset of the kidney rejection timecourse cohort described above for which total leukocyte differentials time-matched to the RNA blood draw were available), acted as an independent **test set** to assess the performance of our deconvolution model. The various datasets used in this analysis are tabulated in **Table 1**.

**Table 1:** Datasets and applications

Dataset	Tissue	Type	Platform	Application	Figures
**GSE28490**	Blood	Isolated leukocyte sub-populations	Affymetrix U133 Plus 2.0	Basis matrix construction	Fig.1A,S1
**GSE28491**	Blood	Isolated leukocyte sub-populations	Affymetrix U133 Plus 2.0	Basis matrix performance in leukocyte isolates	Fig.S2
**GSE20300**	Blood	Peripheral whole blood (PAXgene)	Affymetrix U133 Plus 2.0	Elastic net alpha parameter tuning	Fig.1A
**Heart [22], [26]**	Blood	Peripheral whole blood (PAXgene)	Affymetrix U133 Plus 2.0	Elastic net alpha parameter tuning	Fig.1A
**Kidney [5]**	Blood	Peripheral whole blood (PAXgene)	Affymetrix U133 Plus 2.0	Two-stage, *in silico* deconvolution analysis	Fig.1B,2,3
**Benita ** ***et al*** **. [38]**	Blood	Various	Affymetrix U133A	Tissue-specific enrichment of candidate lists	Fig.3
**Validation**	Blood	Peripheral whole blood (PAXgene)	nanoString nCounter Immunology Assay	Testing lymphocyte-specific ratio hypothesis	Fig.4

### RNA extraction and microarray processing

Blood samples for all subjects and time points were collected in PAXgene tubes and stored at −80°C until analysis. Total RNA was extracted using PAXgene Blood RNA Kits (QIAGEN Inc., Germantown, MD, USA), and integrity and concentration determined using an Agilent 2100 BioAnalyzer (Agilent Technologies Inc., Santa Clara, CA, USA). Affymetrix Human Genome U133 Plus 2.0 (Affymetrix, Inc., Santa Clara, CA, USA) microarrays were processed at the Microarray Core Laboratory at Children's Hospital, Los Angeles in order to assess whole genome expression. The microarrays were checked for quality using the “affy” (version 1.16.0) and “affyPLM” (version 1.14.0) libraries, part of the BioConductor project, as well as “mdqc” (Mahalanobis Distance quality control) [27], an internally developed method. All microarrays that passed quality control were background corrected and normalized using quantile normalization (as in RMA) [28] and summarized using a factor analysis model (factor analysis for robust microarray summarization [FARMS]) [29], via the “farms” library. Finally, we employed the informative/non-informative (I/NI) calls of FARMS to limit our discovery space to internally consistent (at the probe level) probe-sets. The resulting 7820 probe-sets were used as a starting point for all subsequent analyses. Expression datasets obtained from GEO were similarly processed, however FARMS I/NI calls were not applied.

### Two-stage, *in silico* deconvolution analysis

#### Statistical analysis tools

All statistical analyses were performed using the R Statistical Programming Language [30] and a number of packages for the analysis of microarray data included in the Bioconductor project [31]. Feature selection and classification relied on the “glmnet” [32] library, while we inferred cell type proportions using a quadratic programming approach (“limSolve” library) [18]. Plots were created with the excellent “ggplot2” library [33]. The code used to perform the two-stage deconvolution analysis described below will be provided upon request.

#### Modeling mixed expression data

Peripheral whole blood expression was modeled as follows: assume observed expression values *X_ij_* for sample *i = 1, 2, …, n* and genes *j = 1, 2, …, p* measured cell-type proportions *w_ik_* for samples *i = 1, 2, …, n* and cell types *k = 1, 2, …, K*, cell type-specific contribution to the observed gene expression *h_kj_* for cell-types *k* and gene *j*, and a random error term *e_ij_* yields the following equation:
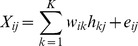



Let *X*, *W*, *H* be matrices with entries *X_ij_* (sample observed expression), *w_ik_* (sample composition), and *h_kj_* (sample cell type-specific contribution to the observed expression) respectively. Having measured *X*, the convolved peripheral whole blood expression, we wish to study *W* and *H* in isolation; that is, we wish to deconvolve *X*.

If *W*, the sample composition, is known, then *H*, the cell type-specific contribution to the observed expression, can be inferred by fitting the above model by regression of each column of *X* on *W*, to yield the coefficients in the corresponding column of *H*, as in csSAM [7]. Fitting the model separately in each group, allows us to interpret the estimated *h_kj_* as the average gene expression for cell-type *k* in the group of samples. The coefficients can then be compared across groups to assess cell type-specific differential expression. Statistical testing employs a permutation scheme to estimate false discovery rate cut-offs. We refer to this as the forward case of deconvolution.

Similarly, *W* can be inferred by fitting the above model by regression of each column of *X* on *H*, to yield the coefficients in the corresponding column of *W*. In this case the cell type-specific contribution to the observed expression, *H*, can be estimated from the expression profiles of isolated cell populations, at least for a minimal subset of genes that exhibit cell type-specific expression. We can then use this estimate of *H*, termed the basis matrix, to deconvolve. The approach was first demonstrated by Lu *et al.*, studying cell-cycle regulation of the yeast *S. Cerevisiae* [16]. Other groups subsequently demonstrated that microarray expression deconvolution can be used to reasonably quantify the constituents of peripheral whole blood [17], [18]. The application of such a computational approach eliminates concerns over collection protocols and time-matching and provides us with a potentially highly granular means of estimating the composition of peripheral whole blood samples. We refer to this as the reverse case of deconvolution.

#### Identifying a dataset suitable for basis matrix construction

The basis matrix for deconvolution is an estimate of the cell type-specific contribution to the observed expression, *H*, for a subset of cell type-specific genes. In the case of peripheral whole blood, this estimate can be obtained from a collection of expression profiles of leukocyte populations isolated from blood. We identified a suitable dataset (GSE28490) on our target platform (Affymetrix U133 plus 2.0) via the Gene Expression Omnibus [34] and chose to include isolated expression profiles for seven relevant leukocyte sub-populations: neutrophils (CD16+CD66b+), eosinophils (CD16-CD66b+), monocytes (CD14+), T cells (CD4+), T cells (CD8+), NK cells (CD56+), and B cells (CD19+).

#### Feature selection, optimal basis matrix construction and prediction of sample composition

Identifying a minimal subset of cell type-specific genes from this collection of isolated leukocyte expression profiles can be framed as a multinomial classification problem with feature selection. Feature selection is necessary because we expect most genes to be non-informative with respect to discriminating between cell types [17]. We assembled a matrix of 54613 probe-sets by seven cell types (neutrophils, eosinophils, monocytes, CD4+ T cells, CD8+ T cells, NK cells and B cells) using the quantile normalized (RMA [28]), log_2_-transformed expression obtained from GSE28490 and fit a multinomial elastic net model [35] (via the “glmnet” library). Probe-sets not present in the kidney rejection timecourse cohort (due to FARMS I/NI calls) were excluded from our feature space using the “exclude” parameter. We similarly excluded probe-sets with low log_2_ fold change between any two cell types by using “limma” [36]. Probe-sets that did not appear in the top 5% in at least one contrast between cell types were added to the “exclude” list. This procedure excluded 53300 probe-sets, leaving 1313 probe-sets eligible for inclusion in the basis matrix. The final number of features to be included was varied by using the elastic net's regularization capabilities. Alpha – the elastic net mixing parameter – was varied between 0 and 1. For each alpha, lambda – the elastic net shrinkage parameter – was set using 10-fold cross-validation via “cv.glmnet” (default) function (“exclude” parameter was set as described above, all other parameters were set to the default value). We selected the largest value of lambda such that the multinomial deviance of the model was within 1 standard error of the minimum. The multinomial deviance was very low across all alphas (0.07–0.09). For all alphas, we obtained the list of features kept in the corresponding model, constructed a basis matrix from their isolated expression profiles and inferred the composition of each sample in our **training** set by reverse deconvolution. We employed a quadratic programming approach (via the “limSolve” library), as in [18] in order to enforce equality/inequality constraints on the model coefficients. Prediction performance was determined by comparing the predicted proportions of neutrophils, lymphocytes, monocytes, and eosinophils to the measured proportions from total leukocyte differentials in this **training** set. Predicted lymphocytes proportions are the sum of the predicted proportions for B cells, CD4+, CD8+ T cells and NK cells. The final basis matrix was that constructed from the largest alpha parameter value (corresponding to a minimal basis matrix) that minimized the root mean squared error (RMSE) in lymphocytes. Performance of the selected matrix was then validated in an independent **test** set.

#### Peripheral whole blood and cell type-specific differential expression analysis

Traditional, two-class differential expression analysis in peripheral whole blood was carried out using Significance Analysis of Microarrays (SAM; via the “samr” library) [37]. Cell type-specific differential expression analysis was performed using csSAM [7], and sample composition either obtained from total leukocyte differentials, or inferred as described above, as input in the non log2-transformed expression data, per [38]. Cell types not detectable in more than 75% of subjects at any given time point were omitted. We use a permissive FDR cutoff of 30% as recommended in [7]. This cell type-specific differential expression analysis was repeated for both a pre-transplant (23 AR, 20 NR; baseline) and post-rejection time point (20 AR, 19 NR) when expression data was available.

#### Peripheral whole blood and cell type-specific gene enrichment analysis

We lacked suitable biological starting material to carry out direct experimental validation of the putative cell type-specific differentially expressed probe-sets identified by csSAM (FDR ≤0.30) in this analysis. In order to establish their plausibility, we assessed both their tissue-specificity and functional enrichment in the context of acute allograft rejection.

The tissue-specificity of all cell type-specific probe-set lists was evaluated in three ways. First, we visualized the median tissue-specific enrichment score (obtained from the Gene Enrichment Profiler database [39]) across the probe-sets that composed each cell type-specific list. Next, tissue specific gene sets were generated from the Gene Enrichment Profiler database. The 99^th^ percentile of the enrichment data across all tissues was used as a threshold. For each tissue, the corresponding gene set was composed of probe-sets with enrichment greater than the threshold value. The tissue-specific enrichment of the candidate cell type-specific gene lists was assessed via a hypergeometric test of their overlap.

This approach was repeated using the MSigDB C7 collection of immunologic signatures and Gene Set Enrichment Analysis (GSEA, [40]). Finally, the functional enrichment of each candidate cell type-specific gene list was similarly assessed using the MSigDB C2 collection of canonical pathway gene sets. For each cell type with detectable cell type-specific differential expression, a cell type-specific ranked list of all probe-sets was generated and submitted to Pre-ranked GSEA. The ranking statistic used is analogous to the “Signal to Noise Ratio” measure that GSEA uses by default and was computed as follows. Recall our model for the convolved mixed expression data:
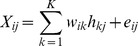



The cell type-specific contribution to the observed expression in the mixed sample expression data, *H*, can be inferred by fitting the above model by regression of each column of *X* on *W*. Fitting the model separately in each group, allows us to interpret the estimated *h_kj_* as the average gene expression for cell-type *k* in the group of samples. The ranking statistic can then be expressed as:
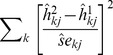



In which 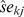 is the estimated standard error of the corresponding difference.

#### Lymphocyte-specific ratio construction

Finally, for all lymphocyte sub-populations where cell type-specific differential expression was present, we mapped the top twenty probe-sets to the nCounter GX Human Immunology Assay. Probe-sets that could be mapped were used to produce a lymphocyte-specific ratio, constructed so as to maximize the difference between AR and NR subjects and overcome the issue of convolution: mean expression of lymphocyte-specific DE genes up-regulated in AR subjects was divided by the mean expression of lymphocyte-specific DE genes down-regulated in AR subjects. This ratio was first evaluated in peripheral whole blood microarray data to determine whether we could, in fact overcome the convolution issue in this manner and, subsequently, tested in samples from an independent cohort whose gene expression was assessed using the nCounter GX Human Immunology Assay.

## Results

### The neutrophil and lymphocyte proportions of peripheral whole blood can be predicted from whole genome expression data using a minimal subset of informative probe-sets

**Figure 1:**
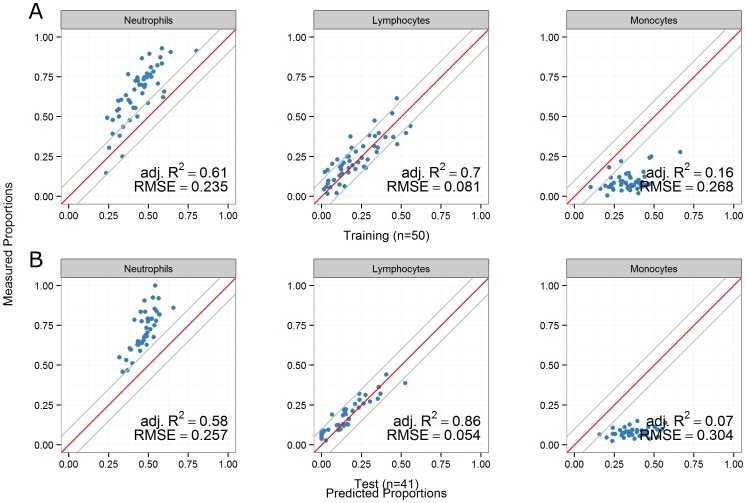
The neutrophil and lymphocyte proportions of peripheral whole blood can be predicted from whole genome expression data using a minimal subset of informative probe-sets. The performance of reverse deconvolution using the optimal basis matrix is assessed by visualizing measured and predicted cell type proportions for neutrophils, lymphocytes and monocytes in **training** (pediatric kidney [n = 24] and heart [n = 26] allograft recipients) and test (kidney allograft recipients [n = 41]) sets of subjects. Predicted lymphocytes proportions are the sum of the predicted proportions for B cells, CD4+, CD8+ T cells and NK cells. Measured and predicted proportions are plotted and the adjusted coefficient of determination (adj. R^2^) and root mean squared error (RMSE) reported in both the **training** (n = 50; **A**) and test sets (n = 41; **B**).

For the selected basis matrix, predicted vs. measured proportions (obtained from total leukocyte differentials), were plotted for neutrophils, lymphocytes and monocytes (which account for >90% of total peripheral whole blood leukocytes) for both our **training** (**Figure 1A**) and independent **test set** (**Figure 1B**) of peripheral whole blood samples. The adjusted R^2^ and root mean squared error (RMSE) are reported. Prediction accuracy was very good in lymphocytes (**training**: R^2^ = 0.70; RMSE  = 0.081; **test**: R^2^ = 0.86; RMSE  = 0.054), but generally poor in neutrophils (**training**: R^2^ = 0.61, RMSE  = 0.235; **test**: R^2^ = 0.58; RMSE  = 0.257) and monocytes (**training**: R^2^ = 0.16; RMSE  = 0.268; **test**: R^2^ = 0.07; RMSE  = 0.304) and eosinophils (**training**: R^2^ = -0.02; RMSE  = 0.032; **test**: R^2^ = 0.29; RMSE  = 0.032; not shown), which are included, but not plotted, in all subsequent analyses.

### Deconvolution of the lymphocyte cellular compartment provides additional insights into the biology of acute kidney allograft rejection

#### Predicted lymphocyte subtype proportions recapitulate the patterns observed in the leukocyte differential data and provide additional information

**Figure 2:**
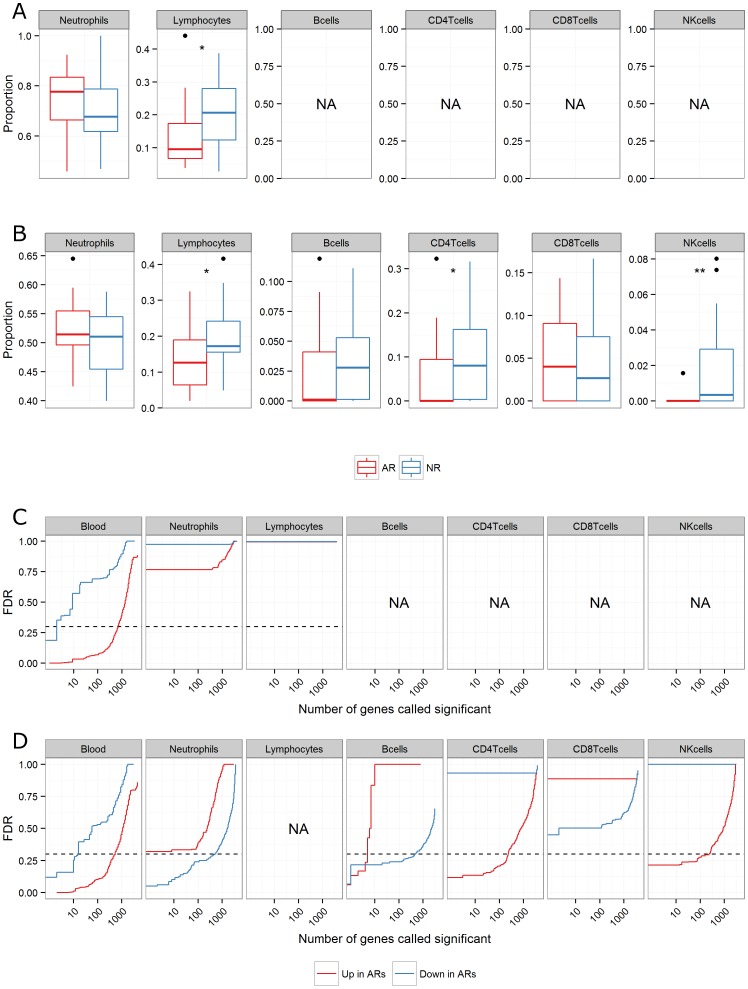
Deconvolution of the lymphocyte cellular compartment provides additional insights into the biology of acute kidney allograft rejection. The cellular composition of peripheral whole blood is plotted for 48 kidney transplant recipients (24AR, 24NR) at the time of a treatable acute rejection episode. Actual cell type proportions were obtained from total leukocyte differentials (time-matched to the RNA collection for the rejection episode), only available for a subset of the 48 subjects (**A**; n = 41, 18AR, 23NR), while predicted cell type proportions were inferred from peripheral whole blood microarray data using the basis matrix from **Figure 1** (**B**; n = 48, 24AR, 24NR). The proportions of all seven cell-types included in the basis matrix are predicted, but only neutrophils and lymphocyte sub-types are shown. Significant differences between groups are labeled (Wilcoxon rank-sum test; p≤0.05 *, p≤0.01 **). Cell type-specific differential expression is assessed using csSAM for 48 kidney transplant recipients (24AR, 24NR) using either actual cell type proportions alone (**C**), or predicted cell type proportions (inferred from peripheral whole blood microarray data) alone (**D**). Cell type-specific differential expression was assessed for all seven cell-types included in the basis matrix, but results are shown only for neutrophils, B cells, CD4+, CD8+ T cells and NK cells (no signal in monocytes, eosinophils). The number of probe-sets called significantly differentially expressed at various false discovery rate (FDR) values is plotted for the one-tailed up and one-tailed down hypotheses (red and blue lines, respectively). A cutoff FDR = 0.30 was selected for discovery purposes (dashed line).

Having established prediction performance, we next applied reverse deconvolution with the selected basis matrix to a cohort of 48 kidney transplant recipients (24AR, 24NR; described above) at the time of a treatable acute rejection episode. The cell type composition of each peripheral whole blood sample was inferred by reverse deconvolution of the mixed expression data to all seven cell types present in the basis matrix (neutrophils, B, CD4+ T, CD8+ T, NK cells, monocytes, and eosinophils; **Figure 2B** - monocytes and eosinophils not shown) and compared to that obtained by total leukocyte differentials (available for 41 subjects, 18AR, 23NR; **Figure 2A**) for AR and NR subjects. Predicted neutrophil and lymphocyte proportions recapitulate the patterns observed in total leukocyte differential data. Predicted neutrophil proportions are lower than expected across all subjects, as previously shown in **Figure 1B**. In both predicted and actual cell type proportion data, lymphocyte proportions are significantly lower in AR compared to NR subjects at the time of rejection. Furthermore, predicted composition data suggests this difference is due to significantly lower CD4+ T-cells and NK-cells in AR compared to NR subjects (Wilcoxon rank-sum test; * p≤0.05; ** p≤0.01, respectively).

#### Cell type-specific differential expression analysis using predicted lymphocyte subtype proportions identifies many B, CD4+ T and NK cell-specific genes associated with acute rejection

Next, cell type-specific differential expression analysis (csSAM[7]) was carried out on peripheral whole blood expression data using compositional data input either obtained from total leukocyte differentials alone (**Figure 2C**; n = 41; 18AR, 23NR) or inferred directly from the peripheral whole blood expression by reverse deconvolution (in a superset of the 41 subjects above; **Figure 2D**; n = 48; 24AR, 24NR). Once again, composition was inferred to all seven cell types present in the basis matrix, but monocyte and eosinophil results are not shown. In each case, the number of probe-sets called as differentially expressed at various false discovery rate (FDR) cutoffs is plotted for the one-tailed up and one-tailed down hypotheses (red and blue lines, respectively). A cutoff FDR ≤30% was selected for discovery purposes (indicated by the dashed line; per recommendation in [7]). There was no statistically significant cell type-specific differential expression between AR and NR subjects when carrying out deconvolution using cell type proportions obtained from total leukocyte differentials (*i.e.*, using neutrophil, lymphocyte, monocyte, and eosinophil proportions as input to csSAM). Repeating the experiment, but substituting in the predicted and summed lymphocyte proportions yielded similar results (not shown). Using the predicted composition data resulted in the identification of 456 probe-sets down-regulated in neutrophils, five probe-sets up- and 445 probe-sets down-regulated in B cells, 221 probe-sets up-regulated in CD4+ T cells and 221 probe-sets up-regulated in NK cells.

### Enrichment analysis of cell type-specific differential expression

**Figure 3:**
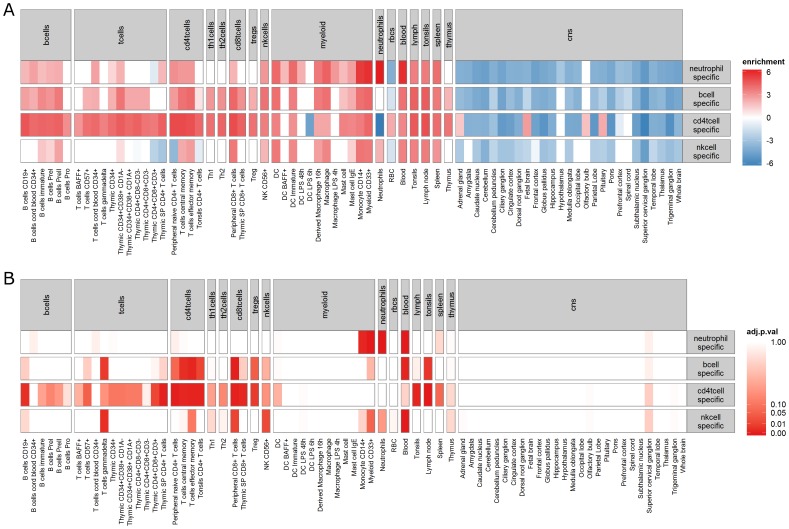
Enrichment analysis of cell type-specific differentially expressed probe-sets establishes their plausibility. The tissue specificity of the cell type-specific gene lists identified in Figure 2 is assessed by visualizing their median enrichment across a wide range of tissues (**A**). Significance of enrichment of each cell type-specific gene list in each tissue is assessed by hypergeometric test (**B**).

In order to evaluate the plausibility of the cell type-specific differentially expressed probe-sets identified by csSAM (FDR ≤0.30) using predicted cell type proportions at the time of a treatable acute rejection episode, we assessed their tissue specificity across a broad range of tissue types[39], and in a more targeted collection of gene sets representing cell states and perturbations within the immune system (Molecular Signatures Database [MSigDB] Collection C7) [40]. We first visualized the relative enrichment [39] of these cell type-specific probe-set lists across a variety of tissues (**Figure 3A**). For each list (neutrophil, B, CD4+ T and NK cell), the median enrichment score across all probe-sets deemed differentially expressed is visualized in a heatmap. The cell type-specific, differentially expressed probe-set lists were generally enriched across all blood tissues compared to CNS in the Benita *et al*. dataset [39]. In addition, the median enrichment was highest in the target tissue for the neutrophil and CD4+ T cell probe-set lists. Differentially expressed probe-sets in B and NK cells were only modestly enriched in the appropriate tissue. Very few of the identified probe-sets had negative enrichment in the appropriate tissue (not shown). Next, we attempted to quantify whether the lists were significantly enriched for any particular tissue by hypergeometric test of their intersection with a collection of tissue-specific gene sets created from the Benita *et al*. dataset (see Methods). The adjusted p-values for these tests (Benjamini-Hochberg FDR [41]) are visualized in a heatmap (**Figure 3B**) and significantly enriched tissues are tabulated in **Table 2**. For the neutrophil, CD4+ T and NK cell lists, the target tissue was significantly enriched (adjusted p = 4.1e-07, 7.2e-05, and 1.8e-02, respectively). Both the neutrophil and NK cell lists appeared to be highly specific, only showing significant enrichment in a few related tissues (neutrophils: blood, myeloid CD33+, monocyte CD14+ and neutrophils; NK cells: blood, T cells gamma-delta, peripheral CD8+ T cells and NK CD56+). Conversely, the B and CD4+ T cell lists were broadly enriched across the B and T lymphocyte tissue types. We note, however, that mature peripheral lymphocyte tissue types were preferentially enriched compared to the immature tissue types (immature, thymic or spleen derived).

**Table 2:** Gene set enrichment of cell type-specific gene lists – Benita *et al.* dataset

Cell Type	Direction	Gene Set	ES	Size	Intersection	Nominal p-val	FDR q-val	FWER p-val
**Neutrophils**	Down	MYELOID CD33+	157.14	138	27	3.77E-15	1.10E-12	1.10E-12
**Neutrophils**	Down	BLOOD	166.17	166	28	7.39E-14	1.08E-11	2.15E-11
**Neutrophils**	Down	NEUTROPHILS	264.63	378	35	5.60E-09	4.09E-07	1.62E-06
**Neutrophils**	Down	MONOCYTE CD14+	120.50	77	10	3.31E-05	6.05E-04	9.18E-03
**B cells**	Down	PERIPHERAL CD8+ T CELLS	34.17	131	15	3.40E-06	1.42E-04	9.73E-04
**B cells**	Down	BLOOD	25.62	166	16	1.85E-05	4.50E-04	5.19E-03
**B cells**	Down	T CELLS EFFECTOR MEMORY	31.12	90	11	2.34E-05	5.26E-04	6.55E-03
**B cells**	Down	T CELLS CENTRAL MEMORY	24.91	106	11	1.20E-04	1.94E-03	3.29E-02
**B cells**	Down	LYMPH NODE	45.61	136	12	3.48E-04	4.85E-03	9.48E-02
**B cells**	Down	T CELLS GAMMADELTA	0.00	74	8	4.93E-04	6.52E-03	1.33E-01
**B cells**	Down	TONSILS CD4+ T CELLS	0.84	52	6	1.13E-03	1.32E-02	3.02E-01
**B cells**	Down	TREG	4.84	65	6	4.14E-03	4.32E-02	1.00E+00
**CD4+ T cells**	Up	LYMPH NODE	64.75	136	14	3.15E-09	3.07E-07	9.14E-07
**CD4+ T cells**	Up	PERIPHERAL NAIVE CD4+ T CELLS	74.73	71	8	1.48E-06	7.22E-05	4.26E-04
**CD4+ T cells**	Up	THYMIC SP CD4+ T CELLS	41.44	82	8	5.03E-06	1.84E-04	1.43E-03
**CD4+ T cells**	Up	PERIPHERAL CD8+ T CELLS	69.63	131	10	6.33E-06	2.05E-04	1.80E-03
**CD4+ T cells**	Up	T CELLS EFFECTOR MEMORY	63.32	90	8	1.09E-05	3.18E-04	3.08E-03
**CD4+ T cells**	Up	TONSILS CD4+ T CELLS	33.22	52	6	1.50E-05	3.97E-04	4.22E-03
**CD4+ T cells**	Up	TONSILS	48.74	180	11	2.53E-05	5.29E-04	7.07E-03
**CD4+ T cells**	Up	THYMIC SP CD8+ T CELLS	39.59	126	9	2.75E-05	5.34E-04	7.63E-03
**CD4+ T cells**	Up	T CELLS CENTRAL MEMORY	73.90	106	8	4.08E-05	7.01E-04	1.13E-02
**CD4+ T cells**	Up	B CELLS CD19+	56.45	125	8	1.47E-04	2.20E-03	4.04E-02
**CD4+ T cells**	Up	TREG	24.46	65	5	5.13E-04	6.52E-03	1.39E-01
**CD4+ T cells**	Up	THYMIC CD4+CD8+CD3+	20.27	77	5	1.26E-03	1.42E-02	3.37E-01
**CD4+ T cells**	Up	T CELLS CD57+	26.97	101	5	4.96E-03	5.00E-02	1.00E+00
**NK cells**	Up	BLOOD	16.06	166	14	4.82E-08	2.82E-06	1.39E-05
**NK cells**	Up	T CELLS GAMMADELTA	0.00	74	6	1.51E-04	2.20E-03	4.11E-02
**NK cells**	Up	PERIPHERAL CD8+ T CELLS	21.35	131	7	1.03E-03	1.25E-02	2.76E-01
**NK cells**	Up	NK CD56+	3.70	110	6	1.66E-03	1.80E-02	4.42E-01

Finally, the csSAM output was used to create a cell type-specific ranking statistic analogous to the Signal to Noise Ratio employed by GSEA, as described previously by our group.[16] For each cell type, all 7820 probe-sets were ranked using this statistic and the resulting cell type-specific probe-set lists submitted to Pre-ranked GSEA using the desktop Java application. This was first performed against the C7 immunologic signatures collection to confirm the tissue specificity results shown above (**Table 3**). It was then repeated with the C2 curated gene set collection's KEGG canonical pathways (**Table 4**). Only gene sets that were significantly enriched (FDR q-value≤0.05), or the top ranking gene sets, are shown for each of the cell type-specific probe-set lists. Few C7 gene sets reached statistical significance in this analysis. For all cell type-specific lists, significantly enriched gene sets (or the top ranked gene set, if none reached significance) were consistent with the inferred cell type origin. Enrichment results in the C2 (REACTOME) gene set analysis were consistent with acute allograft rejection.

**Table 3:** Gene set enrichment of cell type-specific gene lists – MSigDB C7 Immunologic Signatures

Cell Type	Direction	Gene Set	NES	Size	Intersection	Nominal p-val	FDR q-val	FWER p-val
**Neutrophils**	Down	GSE22886_NAIVE_BCELL_VS_NEUTROPHIL_DN	−1.91	153	57	0.00E+00	1.30E-02	1.50E-02
**Neutrophils**	Down	GSE22886_IL2_VS_IL15_STIM_NKCELL_DN	−1.81	42	18	0.00E+00	5.00E-02	1.06E-01
**B cells**	Down	GSE29618_BCELL_VS_MDC_DN	−1.37	121	38	0.00E+00	1.94E-01	3.22E-01
**CD4+ T cells**	Up	GSE3982_CENT_MEMORY_CD4_TCELL_VS_NKCELL_UP	1.85	51	21	0.00E+00	0.00E+00	2.00E-03
**CD4+ T cells**	Up	GSE1460_DP_THYMOCYTE_VS_NAIVE_CD4_TCELL_ADULT_BLOOD_DN	1.72	94	45	0.00E+00	3.00E-02	2.90E-02
**CD4+ T cells**	Up	GSE10325_LUPUS_CD4_TCELL_VS_LUPUS_MYELOID_UP	1.71	114	54	0.00E+00	4.00E-02	3.80E-02
**CD4+ T cells**	Up	GSE3982_EFF_MEMORY_CD4_TCELL_VS_NKCELL_UP	1.70	56	23	0.00E+00	4.00E-02	5.20E-02
**CD4+ T cells**	Up	GSE3982_EOSINOPHIL_VS_CENT_MEMORY_CD4_TCELL_DN	1.69	65	28	0.00E+00	4.00E-02	5.80E-02
**CD4+ T cells**	Up	GSE22886_NAIVE_CD4_TCELL_VS_48H_ACT_TH2_UP	1.68	74	31	0.00E+00	5.00E-02	7.90E-02
**NK cells**	Up	GSE3982_NKCELL_VS_TH1_UP	1.26	44	24	9.00E-03	2.09E-01	9.23E-01

**Table 4:** Gene set enrichment of cell type-specific gene lists – MsigDB C2 KEGG Canonical Pathways

Cell Type	Direction	Gene Set	Function	Size	NES	Nominal p-val	FDR q-val	FWER p-val
**Neutrophils**	Down	NONSENSE MEDIATED DECAY ENHANCED BY EXON JUNCT. COMPLEX	RNA Metabolism	77	−2.06	0.00000	0.00000	0.00000
**Neutrophils**	Down	PEPTIDE CHAIN ELONGATION	RNA Metabolism	71	−2.12	0.00000	0.00000	0.00000
**Neutrophils**	Down	3 UTR MEDIATED TRANSLATIONAL REGULATION	RNA Metabolism	78	−2.14	0.00000	0.00000	0.00000
**Neutrophils**	Down	SRP DEP. COTRANSLATIONAL PROTEIN TARGETING TO MEMBRANE	RNA Metabolism	87	−1.99	0.00000	0.00100	0.01000
**Neutrophils**	Down	TRANSLATION	RNA Metabolism	101	−1.97	0.00000	0.00200	0.01300
**Neutrophils**	Down	RESPIRATORY ELECTRON TRANSPORT	Cell Activation	34	−1.90	0.00000	0.00300	0.03300
**Neutrophils**	Down	METABOLISM OF MRNA	RNA Metabolism	126	−1.88	0.00000	0.00500	0.05500
**Neutrophils**	Down	IMMUNOREGULATORY INTERACTIONS LYMPH. AND NON LYMPH. CELL	Immune Signaling	27	−1.74	0.00800	0.02700	0.34200
**Neutrophils**	Down	INTERFERON ALPHA BETA SIGNALING	Immune Signaling	36	−1.71	0.00000	0.04100	0.49900
**B cells**	Down	MAPK TARGETS NUCLEAR EVENTS MEDIATED BY MAP KINASES	Immune Signaling	15	−1.44	0.00000	0.10100	0.17600
**B cells**	Down	MAP KINASE ACTIVATION IN TLR CASCADE	Immune Signaling	20	−1.39	0.00300	0.13800	0.52800
**B cells**	Down	TGF BETA RECEPTOR SIGNALING ACTIVATES SMADS	Immune Signaling	16	−1.40	0.00700	0.16400	0.44900
**B cells**	Down	SIGNAL TRANSDUCTION BY L1	Immune Signaling	17	−1.37	0.01000	0.16400	0.68500
**B cells**	Down	SIGNALING BY TGF BETA RECEPTOR COMPLEX	Immune Signaling	32	−1.44	0.00000	0.18500	0.16300
**CD4+ T cells**	Up	PEPTIDE CHAIN ELONGATION	RNA Metabolism	71	2.25	0.00000	0.00000	0.00000
**CD4+ T cells**	Up	3 UTR MEDIATED TRANSLATIONAL REGULATION	RNA Metabolism	78	2.24	0.00000	0.00000	0.00000
**CD4+ T cells**	Up	TRANSLATION	RNA Metabolism	101	2.16	0.00000	0.00000	0.00000
**CD4+ T cells**	Up	METABOLISM OF RNA	RNA Metabolism	138	2.03	0.00000	0.00000	0.00000
**CD4+ T cells**	Up	METABOLISM OF PROTEINS	RNA Metabolism	171	1.97	0.00000	0.00000	0.00000
**CD4+ T cells**	Up	TCA CYCLE AND RESPIRATORY ELECTRON TRANSPORT	Cell Activation	58	1.73	0.00000	0.00400	0.08700
**CD4+ T cells**	Up	RESPIRATORY ELECTRON TRANSPORT	Cell Activation	34	1.72	0.00000	0.00500	0.11200
**CD4+ T cells**	Up	RESPONSE TO ELEVATED PLATELET CYTOSOLIC CA2	Immune Signaling	39	1.60	0.00000	0.03300	0.66700
**CD4+ T cells**	Up	IL 2 SIGNALING	Immune Signaling	23	1.58	0.00200	0.03400	0.79400
**CD4+ T cells**	Up	P53 DEPENDENT G1 DNA DAMAGE RESPONSE	Immunoproteosome	29	1.58	0.00400	0.03500	0.78500
**CD4+ T cells**	Up	P53 INDEPENDENT G1 S DNA DAMAGE CHECKPOINT	Immunoproteosome	27	1.58	0.00300	0.03600	0.78400
**CD4+ T cells**	Up	REGULATION OF MITOTIC CELL CYCLE	Immunoproteosome	35	1.57	0.00100	0.03700	0.83600
**CD4+ T cells**	Up	PLATELET ACTIVATION SIGNALING AND AGGREGATION	Immune Signaling	94	1.57	0.00000	0.03700	0.83300
**CD4+ T cells**	Up	IMMUNOREGULATORY INTERACTIONS LYMPH. AND NON LYMPH. CELL	Immune Signaling	27	1.57	0.00200	0.03800	0.85800
**CD4+ T cells**	Up	ANTIGEN PROCESSING CROSS PRESENTATION	Immunoproteosome	45	1.57	0.00200	0.03800	0.85600
**CD4+ T cells**	Up	REGULATION OF APOPTOSIS	Immunoproteosome	29	1.56	0.00400	0.04100	0.88700
**CD4+ T cells**	Up	X-PRESENTATION OF SOLUBLE EXOGENOUS ANTIGENS ENDOSOMES	Immunoproteosome	25	1.54	0.00500	0.04700	0.93600
**CD4+ T cells**	Up	DOWNSTREAM TCR SIGNALING	Immune Signaling	20	1.54	0.01300	0.04700	0.93300
**NK cells**	Up	IRON UPTAKE AND TRANSPORT	Anemia	16	1.44	0.00100	0.02300	0.13600
**NK cells**	Up	RESPONSE TO ELEVATED PLATELET CYTOSOLIC CA2	Immune Signaling	39	1.35	0.00100	0.10800	0.77500
**NK cells**	Up	SEMAPHORIN INTERACTIONS	Immune Signaling	26	1.36	0.00300	0.11600	0.73800
**NK cells**	Up	MHC CLASS II ANTIGEN PRESENTATION	Immune Signaling	35	1.32	0.00300	0.14300	0.93800
**NK cells**	Up	SIGNAL TRANSDUCTION BY L1	Immune Signaling	17	1.33	0.01700	0.15200	0.92900

### Validating a cell type-specific hypothesis in an independent cohort

**Figure 4:**
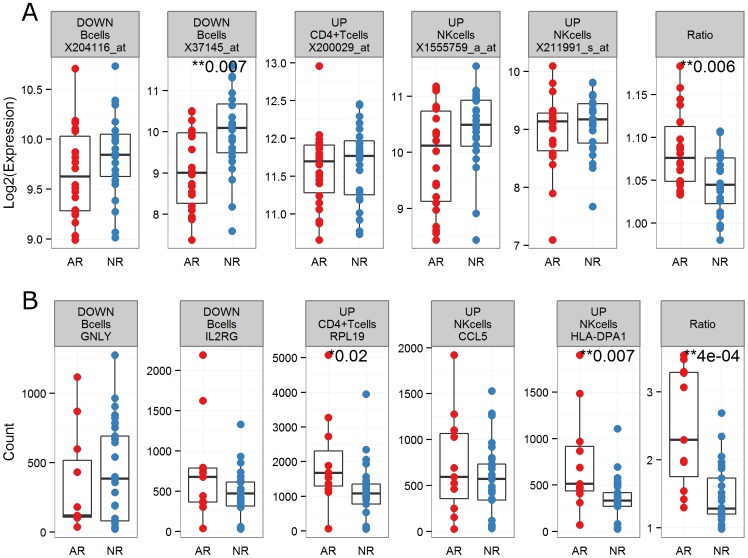
Validating a cell type-specific hypothesis in an independent cohort. The peripheral whole blood expression of top-ranked, cell type-specific differentially expressed genes in B, CD4+ T, and NK cells are plotted for two independent sets of kidney transplant subjects (**A**; 24AR, 24NR; microarray; **B**; 13AR, 31NR; nCounter). A ratio score computed so as to maximize signal between AR and NR subjects, and overcome the convolution issue (see Methods), is also shown. Significant differences between groups are labeled (Wilcoxon rank-sum test; p≤0.05 *, p≤0.01 **).

Peripheral whole blood samples suitable for flow cytometry were never collected for the subjects used in the current study. As a result, experimental validation of the intermediate lymphocyte composition predictions was impossible. Instead, a lymphocyte-specific hypothesis was formulated based on the two-stage deconvolution results (see Methods) and tested in samples from an independent cohort, using a different gene expression assay. A similar approach was recently used to identify a patient's risk of active tuberculosis infection from peripheral whole blood expression data [42]. This lymphocyte specific ratio was significantly up-regulated in AR subjects in the microarray data (**Figure 4A**; Wilcoxon rank-sum test; p = 0.01). We tested our hypothesis in an independent set of subjects using the nCounter technology (**Figure 4B**; Wilcoxon rank-sum test; p = 0.001).

### Kidney acute allograft rejection timecourse

Finally, we wished to demonstrate the power of this approach in enriching existing clinical datasets, by allowing the exploration of cell specific biology without the need for previously collected, time-matched composition information. To this end, we applied deconvolution to study the peripheral whole blood cell type composition (reverse deconvolution) and cell type-specific biology (forward deconvolution) of kidney rejection. For the 48 subjects (24AR, 24NR) from **Figure 2** above, additional PAXgene RNA was available just prior to transplantation (baseline) and after treatable acute rejection (first available time point >seven days after treatable acute rejection; time variable between subjects). We wished to study both cell type composition and cell type-specific expression in these subjects at pre-transplant (baseline), at the time of a treatable acute rejection episode and post-rejection in a simple timecourse experiment.

#### Deconvolution of the lymphocyte compartment of peripheral whole blood elucidates patterns of differential cell type composition of peripheral whole blood between AR and NR subjects before, during and after an episode of treatable acute kidney allograft rejection

**Figure 5:**
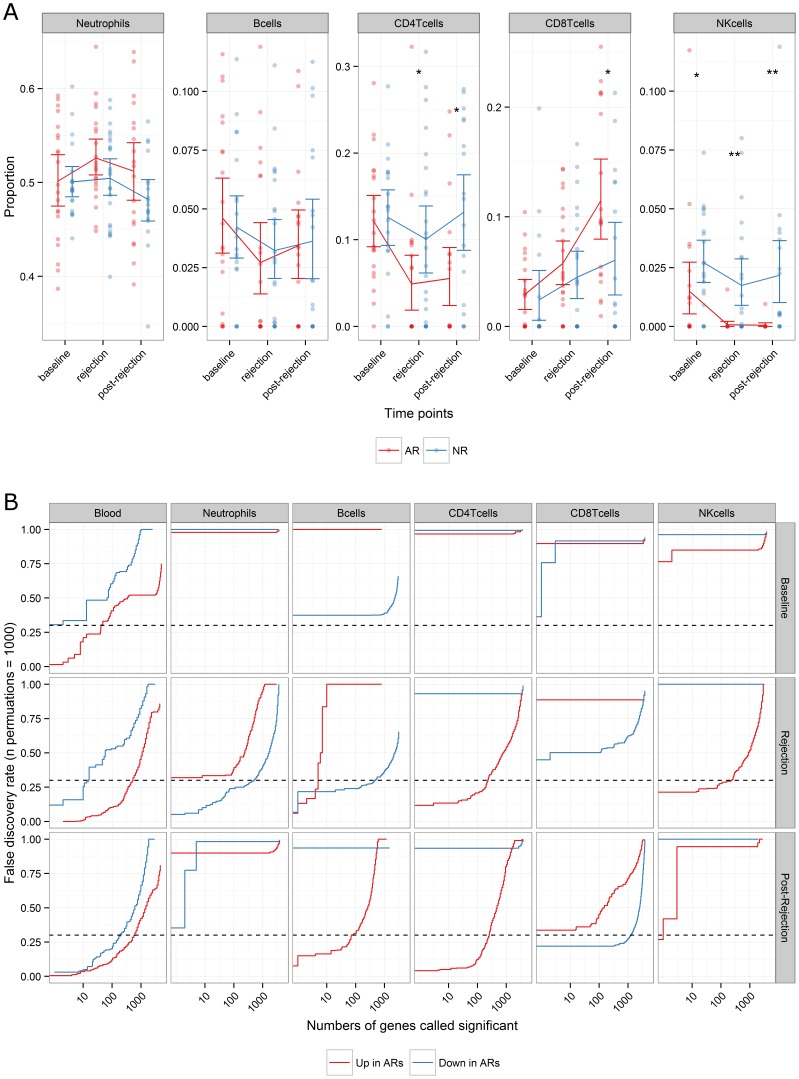
Deconvolution of the lymphocyte compartment of peripheral whole blood elucidates patterns of differential composition, and differential cell type-specific gene expression, between AR and NR subjects before, during and after an episode of treatable acute kidney allograft rejection. The composition of the peripheral whole blood samples was inferred from mixed expression data for all 48 subjects (24 AR, 24 NR) at the time of a treatable acute rejection episode, as well as at baseline (23 AR, 20 NR) and after rejection had resolved (20 AR, 19 NR) when expression data was available (**A**). The mean (and bootstrapped confidence intervals) of the proportions of neutrophils, B cells, CD4+, CD8+ T cells and NK cells are plotted for each group, at each time point. Significant differences between groups are labeled (Wilcoxon rank-sum test; p≤0.05 *, p≤0.01 **). Cell type specific differential expression analysis (csSAM) was performed using the sample composition information inferred above (**B**). Cell type-specific differential expression was assessed for all seven cell-types included in the basis matrix, but results are shown only for neutrophils, B cells, CD4+, CD8+ T cells and NK cells (no signal in monocytes, eosinophils). Cell types not detectable in more than 75% of subjects at a given time point were omitted from the model. For each time point, the number of probe-sets called significantly differentially expressed at various false discovery rate (FDR) values is plotted for the one-tailed up and one-tailed down hypotheses (red and blue lines, respectively). A cutoff FDR ≤0.30 was selected for discovery purposes (dashed line).

The composition of the peripheral whole blood samples was inferred from mixed expression data [16], [17] for all 48 subjects (24 AR, 24 NR) at the time of a treatable acute rejection episode, as well as at baseline (23 AR, 20 NR) and after rejection had resolved (20 AR, 19 NR) when expression data was available (**Figure 5A**). The mean (and bootstrapped confidence intervals) of the proportions of neutrophils, B cells, CD4+, CD8+ T cells, and NK cells are plotted for each group, at each time point. Significant differences between groups are labeled (Wilcoxon rank-sum test; p≤0.05 *, p≤0.01 **). Statistically significant differences in the mean proportions between groups were observed in the following cell types, at the following time points: at baseline, NK cells were depressed in AR subjects; at the time of rejection, CD4+ T cells and NK cells were depressed in AR subjects; post-rejection, CD8+ T cells were elevated in AR subjects, while CD4+ T cells and NK cells remained depressed.

#### Deconvolution of the lymphocyte compartment of peripheral whole blood elucidates patterns of differential cell type-specific transcriptional activity between AR and NR subjects before, during and after an episode of treatable acute kidney allograft rejection

Next, cell type-specific differential expression analysis was performed using the inferred sample composition obtained above for all 48 subjects (24 AR, 24 NR) at the time of a treatable acute rejection episode, as well as at baseline (23 AR, 20 NR) and after rejection had resolved (20 AR, 19 NR) when expression data was available. The number of probe-sets called differentially expressed at various FDR cutoffs is plotted for the one-tailed up and one-tailed down hypotheses (red and blue lines, respectively; **Figure 5B**). A cutoff FDR ≤30% was selected for discovery purposes (indicated by the dashed line). Cell types not detectable in more than 75% of subjects at a given time point were omitted. In peripheral whole blood, 40 probe-sets were identified as differentially expressed at baseline (all up-regulated in AR), 506 probe-sets (mostly up-regulated in AR) were identified at the time of rejection and 735 probe-sets (up- and down-regulated in AR) post-rejection. As expected, there was no statistically significant cell type-specific signal at baseline. Hundreds of probe-sets were identified as differentially expressed both at rejection and post-rejection in various cell sub-populations. At the time of rejection, 456 probe-sets were down-regulated in neutrophils and 445 in B cells in ARs, while 221 probe-sets were up-regulated in CD4+ T cells and 221 in NK cells in ARs. Cell type-specific signal in neutrophils and NK cells resolved post-rejection, but persisted in CD4+ T cells (243 probe-sets up-regulated, 83 in common with the rejection time point; Chi-Square Test: p<2.2e-16). The signal in B cells was radically modified: from nearly 500 probe-sets being down-regulated at the time of rejection to 70 probe-sets being up-regulated in AR subjects post-rejection (2 probe-sets in common, both mapping to WD repeat domain 45; Chi-Square Test: p = 0.4892). In addition, nearly 1200 probe-sets were down-regulated in CD8+ T cells in ARs.

## Discussion

Clearly, cell type-specific expression is of interest when studying complex tissues. Experimental separation techniques that could facilitate study of the various components of complex tissues exist, but practical considerations limit their use in clinical or translational research settings. Statistical forward deconvolution is an alternative [7], but requires that relevant composition information be available for every sample: time-matched to the RNA collection, sufficiently granular and accurate. This limits its utility. Retrospective study of existing expression data by this approach will be unfeasible in most cases. In a clinical setting, total leukocyte differentials have been proposed as an affordable source of composition information [7], however they offer insufficient granularity in practice. Flow cytometry can provide much higher granularity, but significantly increases the complexity of sample collection and processing protocols and results in additional costs. Statistical reverse deconvolution may be used to infer the composition of complex tissue samples from their expression data by using isolated expression profiles of a subset of informative genes [16]–[18]. The purpose of this study was to demonstrate both the feasibility of, and the additional utility provided by, a two-stage, *in silico* deconvolution of the lymphocyte compartment of peripheral whole blood expression data to study both the composition of, and the diverse cell type-specific expression programs at play in, this complex tissue.

### Basis matrix construction and composition prediction performance

We first evaluated the previously published Abbas *et al*. basis matrix [17], but found its performance inadequate in our data (not shown). The reasons for this were unclear, but might be related to platform scaling issues (both basis matrix and deconvolved samples from Abbas *et al.* were processed on the U133A platform and applying our own basis matrix in GeneST1.1 data resulted in poor prediction performance; not shown). We therefore elected to construct our own U133 plus 2.0-based basis matrix, in order to study the cell types we deemed interesting in this context. A suitable collection of the isolated expression profiles of various leukocyte sub-populations was identified on the GEO website and an optimal basis matrix constructed by fitting a multinomial elastic net model to the isolated expression profiles of seven leukocyte sub-populations. This was performed in the RMA normalized, log2-transformed data (the effect of normalization and log_2_-transformation of the raw expression data on prediction performance were explored; see **Figures S3 and S4**). The size of the resultant basis matrix was tuned to maximize prediction performance of reverse deconvolution for lymphocytes in a training set of 50 heart and pediatric kidney allograft recipients for which total leukocyte differentials time-matched to the RNA blood draw were available. We observed no bias in prediction performance between subjects either undergoing acute allograft rejection or not (data not shown). The selected basis matrix was then used to predict the blood leukocyte composition of samples from 48 kidney allograft recipients using reverse deconvolution. We confirmed our ability to predict lymphocyte proportions in a subset of these 48 subjects for which total leukocyte differentials time-matched to the RNA blood draw were available. These 41 subjects acted as an independent test set to assess the prediction performance of the selected basis matrix.

Prediction performance in lymphocytes was comparable to current state of the art total error rate for lymphocyte measurement by total leukocyte differentials (Prediction RMSE  = 5.4–8.1%; total leukocyte differential lymphocyte measurement error: 4.0–11.9% [43]). Performance in neutrophils was worse, with a marked increase in bias at higher measured neutrophil proportions. Performance in monocytes was poor, but no worse than the typical measurement error rate of total leukocyte differentials when quantifying these cell sub-populations (13.4–58.7%, respectively [43]). We note that monocyte prediction performance bias mirrored that observed in neutrophils. To test whether this was a result of the conditional negative correlation between cell type proportions, we ranked samples by their absolute error in neutrophils and monocytes, compared these lists and found no statistically significant difference between them (Wilcoxon rank-sum test; p = 0.10). This suggests that the basis matrix as constructed may be misattributing neutrophil- and monocyte-specific signals. Storage conditions (room temperature or refrigerated), precise collection time and time elapsed from blood draw to processing for each sample have been shown to result in changes in peripheral whole blood composition [44]. Differences in the collection time and time to process blood drawn for hematology analysis and that destined for RNA extraction could contribute to the observed bias, for example. The use of PAXgene blood tubes enabled cellular RNA to be rapidly protected from degradation, post blood-draw, for the peripheral whole blood expression profiles. The cellular RNA of the isolated leukocyte profiles that form the basis matrix (GSE28490) were not similarly protected. That neutrophils are affected is perhaps unsurprising: they are fragile, autolytic and cannot be preserved [45].

Varying the composition of the basis matrix did not negatively affect prediction accuracy in lymphocytes (**Figure S1**). The elastic net alpha parameter was tuned from 1 to 0 and RMSE of the predicted lymphocyte proportion was computed as the number of genes included in the basis matrix increased. We observed that, for most values of alpha (0.2≤α≤0.8; 442≥ number of genes ≥94), the RMSE was ≤10%. Predicted neutrophil proportions were similarly robust (not shown). An initial concern was that basis matrix genes might be under differential regulation in the perturbed state of interest. The similar (and robust to gene membership) prediction performance in both AR and NR subjects in both our training and test cohort suggests that the feature selection strategy we adopted resulted in an unbiased basis matrix.

### Predicted lymphocyte subtype proportions during acute kidney allograft rejection

Having selected an optimally performing basis matrix, we wished to demonstrate the utility of this approach in studying a complex biological process. We studied the proportions of various lymphocyte sub-populations during kidney allograft rejection in the inferred composition data. Our results were consistent with a previously published flow cytometry analysis of the peripheral blood of kidney transplant recipients by Sagoo *et al*. [46] In that study, both B and NK cell proportions were found to be elevated in the peripheral whole blood of tolerant kidney transplant recipients. While CD4+ T cells were found to be relatively less abundant in the peripheral blood of NR subjects in the Sagoo *et al*. study, these were activated CD3+CD4+ T cells and not strictly comparable to the un-activated sub-population quantified here. It is conceivable that both observations are measuring the same phenomenon, namely increased activation of CD4+ T cells in AR subjects. This should result in comparatively lower activated CD3+CD4+ T cells proportions in NR subjects and, conversely, higher proportions of un-activated CD4+ T cells in these same subjects when compared to ARs. Functionally, the lower peripheral blood (marginal) B, CD4+ T and NK cell proportions in AR subjects could be the result of increased infiltration of these cell types into the allograft, which would be consistent with our current understanding of solid organ rejection, in which NK cells act as facilitators of solid organ rejection, amplifying early graft inflammation and supporting the activity of alloreactive T cells [47]–[49]. The results of GSEA carried out on the ranked CD4+ T and NK cell-specific probe-set lists are consistent with this hypothesis: the KEGG leukocyte transendothelial migration gene set, as well as many inflammatory gene sets, were enriched in circulating CD4+ T and NK cells. No other statistically significant differences in the relative cell type abundances were observed.

### Cell type-specific differential gene expression during acute kidney allograft rejection

The inferred composition estimates were then used as input to csSAM. The inclusion of phenotypically homogeneous and biologically relevant lymphocyte sub-populations provides more useful context when interpreting cell type-specific differential expression results. More importantly, deconvolving to more phenotypically homogeneous components should improve the sensitivity of the approach (by satisfying the model's underlying assumption of phenotypically homogeneous linear components).

Validating the results of statistically powered cell type-specific differential expression analysis is generally challenging because experimental separation protocols are known to result in modified gene expression [14], [15]. In addition, we lacked the necessary biological material to carry out true experimental validation (FACS and separate expression profiling in each of the separated cell type isolates). Consequently, we were unable to study precisely how the use of computationally derived cell composition estimates affected the sensitivity of the cell type-specific differential expression analysis. Because csSAM does not explicitly account for errors in the composition estimates used as input, or control for these errors when estimating FDR cutoffs, demonstrating plausibility of the cell type-specific differentially expressed probe-set lists was crucial. This is a concern for cell type-specific differential expression results derived from any compositional estimate (including, *e.g.*, total leukocyte differentials).

To evaluate the plausibility of the derived cell type-specific probe-set lists, we turned to tissue and functional enrichment strategies, as well as the literature. We first observed that the cell type-specific probe-set lists are distinct, though some lists exhibit significant overlap (**Figure S5**). Lists that overlap significantly between cell types with opposite directionality (*e.g.*, down in neutrophils and up in NK cells), serve to highlight the issue of convolution in peripheral whole expression data. Tissue specific enrichment results, using both the Benita *et al*. and the MSigDB C7 collection of immunologic gene sets, were consistent with the cell type origin. Hypergeometric test of the overlaps between tissue specific gene sets and the cell type-specific probe-set lists in the Benita *et al*. data suggest that both the neutrophil and NK cell lists were very cell type-specific, while the B and CD4+ T cell lists were more broadly representative of mature, circulating B or T lymphocytes. While no C7 gene sets were significantly enriched in the B cell list, the CD4+ T cell list was highly specific for CD4+ T cell states and perturbations. Tissue-specific enrichment results thus suggest that our two-stage, *in silico* deconvolution strategy is yielding truly cell type-specific probe-sets.

Furthermore, functional enrichment results using the MSigDB C2 (REACTOME) collection of gene sets were consistent with our current understanding of solid organ rejection, in which NK cells act as facilitators, amplifying early graft inflammation and supporting the activity of alloreactive T cells [47]–[49]. Many pathways related to immune signaling were either significantly enriched (FDR≤0.05) or trending in these cell populations, including platelet-mediated activation pathways, which have been implicated in recruitment of T lymphocytes in allograft rejection [50]. Interestingly, while enriched in both CD4+ T and NK cells, the genes contributing to this enrichment were distinct (not shown). In addition, the IL2 signaling pathway, a regulatory hub of allograft rejection and target of many immunosuppressive therapies [51], as well as many pathways related to RNA and protein metabolism (transcription, translation) and antigen processing and presentation (immunoproteosome) were enriched in CD4+ T cells only. Conversely, RNA metabolism (transcription, translation) and immune signaling are depressed in neutrophils. This highlights the issue of convolution of signal in peripheral whole blood expression data. No C2 (REACTOME) gene sets reached significance in B cells. However, our results at the gene level are consistent with the Sagoo *et al*. study: six of the ten most significant differentially expressed genes in that study were identified *post facto* as B cell specific and down-regulated in AR subjects. That study also found that B cells from NR subjects (tolerant) had skewed cytokine response, with a higher propensity for TGF-β production than B cells from AR subjects. TGF-β was found to be down-regulated in B cell in AR subjects and TGF-β signaling was one of the most negatively enriched gene sets in these subjects, though it did not reach statistical significance.

Having established plausibility, we attempted to validate some of these cell type-specific results in an independent patient cohort using the nCounter GX Human Immunology Assay. A lymphocyte-specific ratio for each patient was computed by taking the mean expression of probe-sets up-regulated in CD4+ T and NK cells in AR subjects at the time of rejection and dividing it by the mean expression of probe-sets down-regulated in B cells in AR subjects at the time of rejection, for probe-sets that could be mapped to the nCounter assay. The ratio was constructed so as to maximize the difference between AR and NR subjects. It was significantly different between AR and NR subjects when computed from the peripheral whole blood microarray data (24AR, 24NR), demonstrating it could overcome the convolution issue in peripheral whole blood expression data, and this result was replicated on the nCounter platform, in independent samples. While it is likely that the cell type-specific lists generated by this two-stage deconvolution approach include false positives, this replication result demonstrates that it can be a fruitful strategy, particularly if the goal is hypothesis generation.

### Two-stage, *in silico* deconvolution of the lymphocyte compartment applied to a timecourse study of acute kidney allograft rejection

Finally, we wished to demonstrate the utility of this approach by applying it to a timecourse study of acute kidney allograft rejection. Such a study would have been extremely challenging to implement had we been reliant on the availability of time-matching of the RNA blood draw (PAXgene tube) to total leukocyte differentials (EDTA whole blood tube) to assess sample composition. It is presented below as an illustrative example of the kind of retrospective analysis that a two-stage, *in silico* deconvolution strategy enables using existing clinical samples.

The pre-transplant time point (baseline) is included as a control. We expect both groups to be similar at this time. The identification of approximately 50 differentially expressed probe-sets in the peripheral whole blood analysis, including eight at FDR ≤10% and three probe-sets at FDR ≤5%, serves to reinforce that peripheral whole blood expression data can be convolved by differences in composition across subjects. Conversely, no statistically significant, cell type-specific, differential expression is identified between AR and NR subjects at this pre-transplant time point. The composition of peripheral whole blood from AR and NR subjects is also comparable pre-transplant, with the exception of NK cell proportions, which are significantly lower in AR subjects. Though this may simply be an artefact of inaccurate prediction, NK cells have been previously reported to facilitate the induction of tolerance [47]. It is plausible that low circulating NK cell proportions may result in disrupted induction of tolerance in AR subjects.

Post-rejection, the cell type-specific signal seen at the time of rejection resolves in neutrophils and NK cells. It is interesting that these cell types, both involved with the early, innate immune response [47], appeared to resolve rapidly post-rejection. Conversely, the rejection time point signal persisted, at both the probe-set (83 up-regulated are in common with the rejection time point; Chi-Square Test: p<2.2e-16) and gene set level (not shown), in CD4+ T cells. This is accompanied by lower CD4+ T cell proportions in circulating blood, possibly as a result of continued infiltration of these cells into the allograft, or as a result of increased immunosuppressive load in AR subjects. The elevated CD8+ T cell proportions observed in AR subjects post-rejection is accompanied by down-regulation of nearly 1200 probe-sets in these same cells. No clear enrichment signal arises, however (KEGG ribosome and amyotrophic lateral sclerosis gene sets are enriched in AR subjects). The drastic changes in the B cell compartment are similarly difficult to interpret. This may be due to the majority of the signal at this time point being treatment-, rather than disease-, driven and thus poorly summarized by gene sets in the KEGG pathways collection.

### Limitations

The current study has two main limitations. First, while neutrophil and monocyte prediction performance can be directly validated using total leukocyte differential data, predictions for the various lymphocyte sub populations cannot. The validity of applying reverse deconvolution using the selected basis matrix to peripheral whole blood was assessed by experimenting with expression profiles obtained from leukocyte sub populations isolated from peripheral whole blood (distinct from those used to construct the basis matrix; GSE28491). These profiles were appropriately deconvolved (**Figure S2**), confirming that our selected basis matrix can accurately predict the proportions of pure leukocyte populations isolated from peripheral whole blood. However, absent more granular independent measurement of composition we are unable to assess the prediction accuracy at the lymphocyte sub-population level (B cells, NK cells and CD4+ and CD8+ T cells) in mixtures. This is an important limitation of the current work, which we hope to address in future studies (*e.g.*; by using flow cytometry to quantify the sub-populations of interest). Unfortunately, the required biological materials were not available for the subjects used in this study. Sample composition was not, however, the primary focus of the current work. Rather we wished to study cell type-specific differential expression in lymphocyte sub-populations before, during and after acute rejection of a renal allograft. Prediction error in the composition estimates used as input for this analysis, though undesirable, is carried forward into the cell type-specific differential expression results, potentially leading to an inflated rate of false positives. We also note that the csSAM procedure does not account for errors in the composition estimates used as input when estimating FDR cutoffs. Ultimately, any specific hypotheses based on the cell type-specific differentially expressed gene lists would need to be validated in independent samples and using a different technology to assay gene expression.

Second, validation of the cell type-specific differentially expressed probe-sets identified by csSAM is challenging in practice because it is likely that experimental separation affects expression [14], [15]. Instead of direct validation, we attempted to establish plausibility for the various cell type-specific gene lists produced using various tissue and functional enrichment strategies, and existing literature on kidney allograft rejection. In addition, we were able to validate a small subset of these lymphocyte-specific differentially expressed genes in independent samples using the nCounter assay. This was achieved by computing a ratio designed to overcome the convolution issue and demonstrates that computational approaches such as the one described here can provide valuable insights, particularly when the goal is hypothesis generation. Forward deconvolution in peripheral whole blood could be validated in general, for example by separating and quantifying the components cell types of peripheral whole blood samples (*e.g.*; using fluorescence-activated cell sorting) under different experimental conditions and assessing expression in these isolated components, as well a reconstituted, equal volumes mixture of these components. The cell type-specific expression can then be obtained by deconvolution of the reconstituted mixture expression data using the measured proportions of the components in the original sample and compared to that observed in the isolated components. This is analogous to the *in silico* proof of concept originally presented by Shen-Orr *et al*. [7] Implementing such a scheme in the context of a large, multi-centre, clinical trial is daunting and presents serious practical challenges, which only serves to highlight the importance of continued research into statistical deconvolution approaches.

### Conclusion

The two-stage, *in silico* deconvolution approach described here has allowed us to deconvolve the lymphocyte compartment of peripheral whole blood and study both the cell type composition of, and cell type-specific expression in, existing clinical samples. This did not require the collection of time-matched sample composition information. The inferred composition data provided a more informative context for interpretation of the cell type-specific differential expression results. The predicted cell type proportion and cell type-specific differential expression results at the time of rejection were consistent with the experimental context, consistent with previously published work, and suitable for exploratory analysis and hypothesis generation. Finally, we demonstrated the power of this approach in allowing us to gain additional insight from existing clinical samples for which only peripheral whole blood expression data is available. While we focused on acute allograft rejection in kidney transplantation, this two-stage, *in silico* deconvolution approach should allow for similar studies to be carried out in any peripheral whole blood expression datasets on the Affymetrix U133 Plus 2.0 platform, allowing for re-examination of more than 80000 arrays, comprising nearly 3000 experiments on GEO alone. The approach should be broadly applicable to the study of any complex tissue for which isolated component expression profiles exist on the same technology platform, including next generation sequencing, and may prove a fruitful strategy in many contexts.

## Supporting Information

Figure S1 **Robustness of the basis matrix.** The condition number (kappa) of the basis matrix and root mean squared error (RMSE) of the predicted lymphocyte proportion in AR and NR subjects is plotted as alpha, the elastic net tuning parameter, is adjusted between 1 and 0. Corresponding number of genes included in the basis matrix thus constructed is indicated.
(TIFF)

Figure S2 **Performance of expression deconvolution on purified leukocytes supports using it on peripheral whole blood.** Reverse deconvolution of an independent test set of leukocytes isolated from peripheral whole blood (GSE28491) demonstrates that various cell types are accurately deconvolved. Plotted data is the predicted proportion of that cell type in the whole sample produced by reverse deconvolution of each of seven purified cell type expression profiles. Data points are from independent subject samples.
(TIFF)

Figure S3 **Reverse deconvolution is more accurate when data is quantile normalized.** The performance of reverse deconvolution using the optimal basis matrix is assessed by visualizing measured and predicted cell type proportions for neutrophils, lymphocytes and monocytes in the **training** set (pediatric kidney [n = 24] and heart [n = 26] allograft recipients), either quantile normalized (**A**) or not (**B**). Predicted lymphocyte proportions are the sum of the predicted proportions for B cells, CD4+, CD8+ T cells and NK cells. Measured and predicted proportions are plotted and the adjusted coefficient of determination (adj. R^2^) and root mean squared error (RMSE) reported.
(TIFF)

Figure S4 **Reverse deconvolution is more accurate when data is log_2_-transformed.** The performance of reverse deconvolution using the optimal basis matrix is assessed by visualizing measured and predicted cell type proportions for neutrophils, lymphocytes and monocytes in both the **training** (pediatric kidney [n = 24] and heart [n = 26] allograft recipients) and **test** (kidney allograft recipients [n = 41]) sets, either log_2_-transformed (top) or not (bottom). Predicted lymphocyte proportions are the sum of the predicted proportions for B cells, CD4+, CD8+ T cells and NK cells. Measured and predicted proportions are plotted and the adjusted coefficient of determination (adj. R^2^) and root mean squared error (RMSE) reported.
(TIFF)

Figure S5 **Overlap between the various cell type-specific differentially expressed probe-set lists at the time of rejection.** A Venn diagram showing the overlap between the various cell type-specific differentially expressed probe-set lists obtained in **Figure 2**.
(TIFF)

Table S1 **Subject Demographics.**
(XLS)
