# Supplementary figures and images for: Two-Stage, In Silico Deconvolution of the Lymphocyte Compartment of the Peripheral Whole Blood Transcriptome in the Context of Acute Kidney Allograft Rejection
Source: PLoS One. 2014 Apr 14;9(4):e95224. doi: 10.1371/journal.pone.0095224 (PMC3986379; doi:10.1371/journal.pone.0095224)

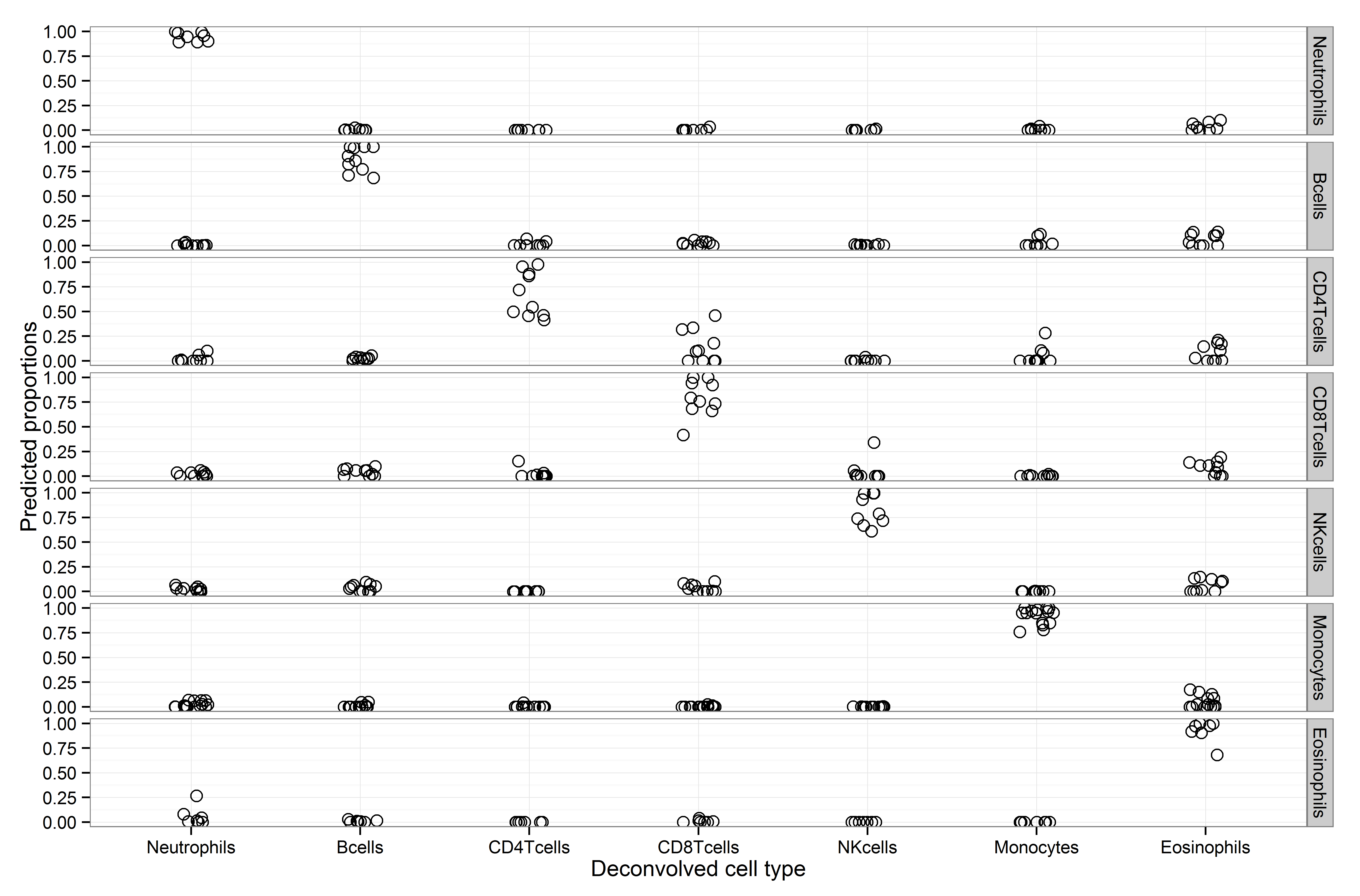

Supplement: Figure S2 — Performance of expression deconvolution on purified leukocytes supports using it on peripheral whole blood. Reverse deconvolution of an independent test set of leukocytes isolated from peripheral whole blood (GSE28491) demonstrates that various cell types are accurately deconvolved. Plotted data is the predicted proportion of that cell type in the whole sample produced by reverse deconvolution of each of seven purified cell type expression profiles. Data points are from independent subject samples. (TIFF) [file pone.0095224.s002.tiff]

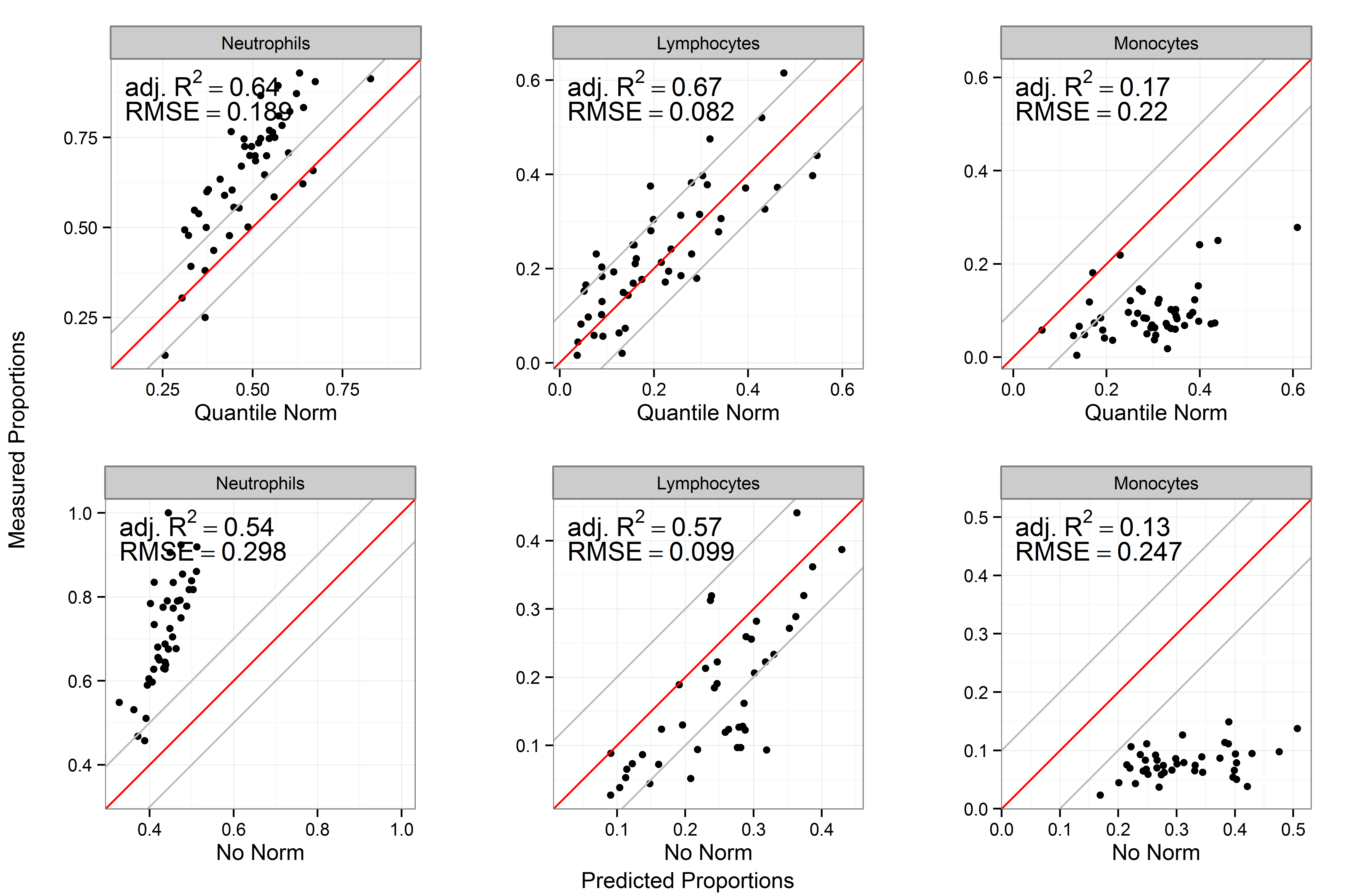

Supplement: Figure S3 — Reverse deconvolution is more accurate when data is quantile normalized. The performance of reverse deconvolution using the optimal basis matrix is assessed by visualizing measured and predicted cell type proportions for neutrophils, lymphocytes and monocytes in the training set (pediatric kidney [n = 24] and heart [n = 26] allograft recipients), either quantile normalized (A) or not (B). Predicted lymphocyte proportions are the sum of the predicted proportions for B cells, CD4+, CD8+ T cells and NK cells. Measured and predicted proportions are plotted and the adjusted coefficient of determination (adj. R2) and root mean squared error (RMSE) reported. (TIFF) [file pone.0095224.s003.tiff]

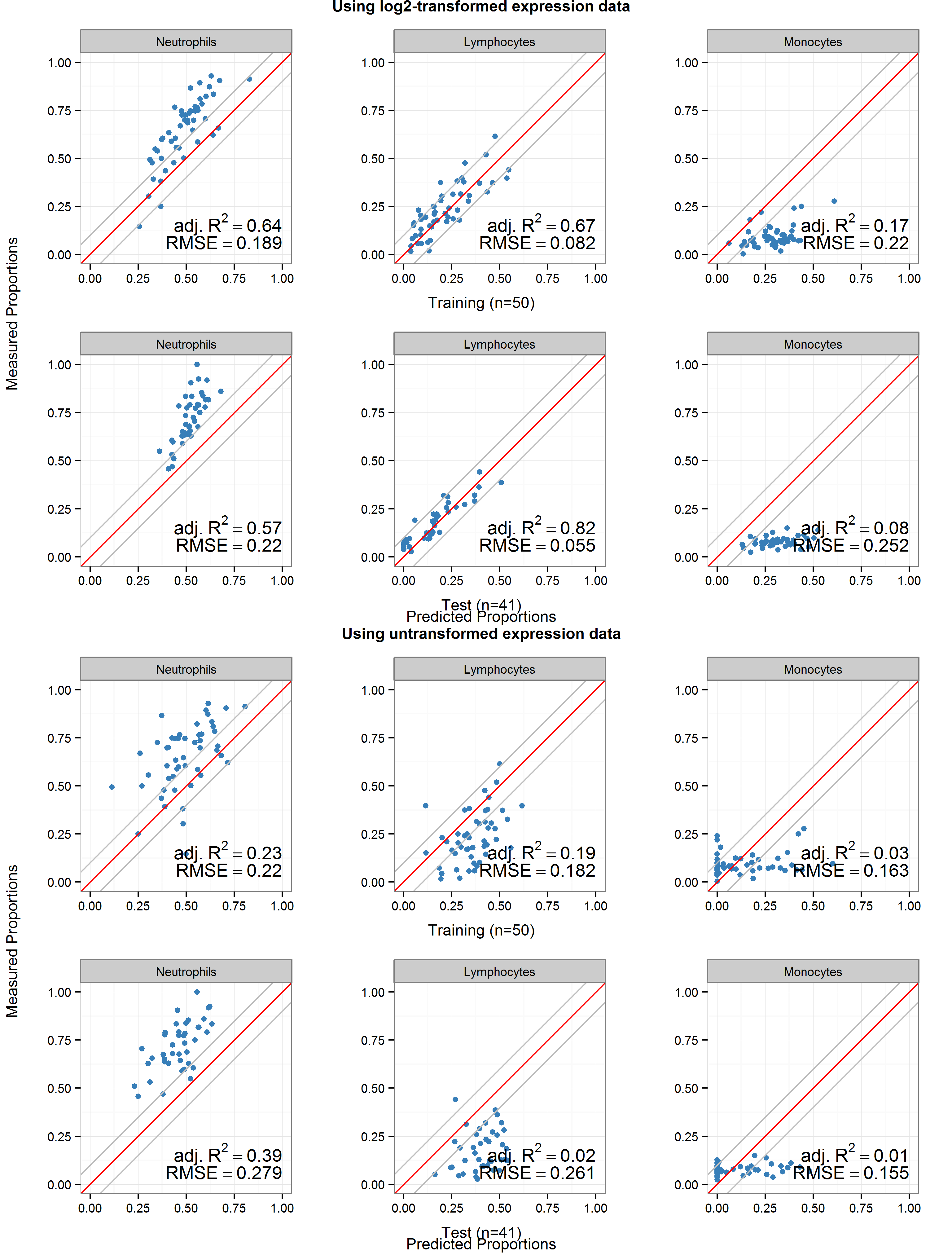

Supplement: Figure S4 — Reverse deconvolution is more accurate when data is log2-transformed. The performance of reverse deconvolution using the optimal basis matrix is assessed by visualizing measured and predicted cell type proportions for neutrophils, lymphocytes and monocytes in both the training (pediatric kidney [n = 24] and heart [n = 26] allograft recipients) and test (kidney allograft recipients [n = 41]) sets, either log2-transformed (top) or not (bottom). Predicted lymphocyte proportions are the sum of the predicted proportions for B cells, CD4+, CD8+ T cells and NK cells. Measured and predicted proportions are plotted and the adjusted coefficient of determination (adj. R2) and root mean squared error (RMSE) reported. (TIFF) [file pone.0095224.s004.tiff]

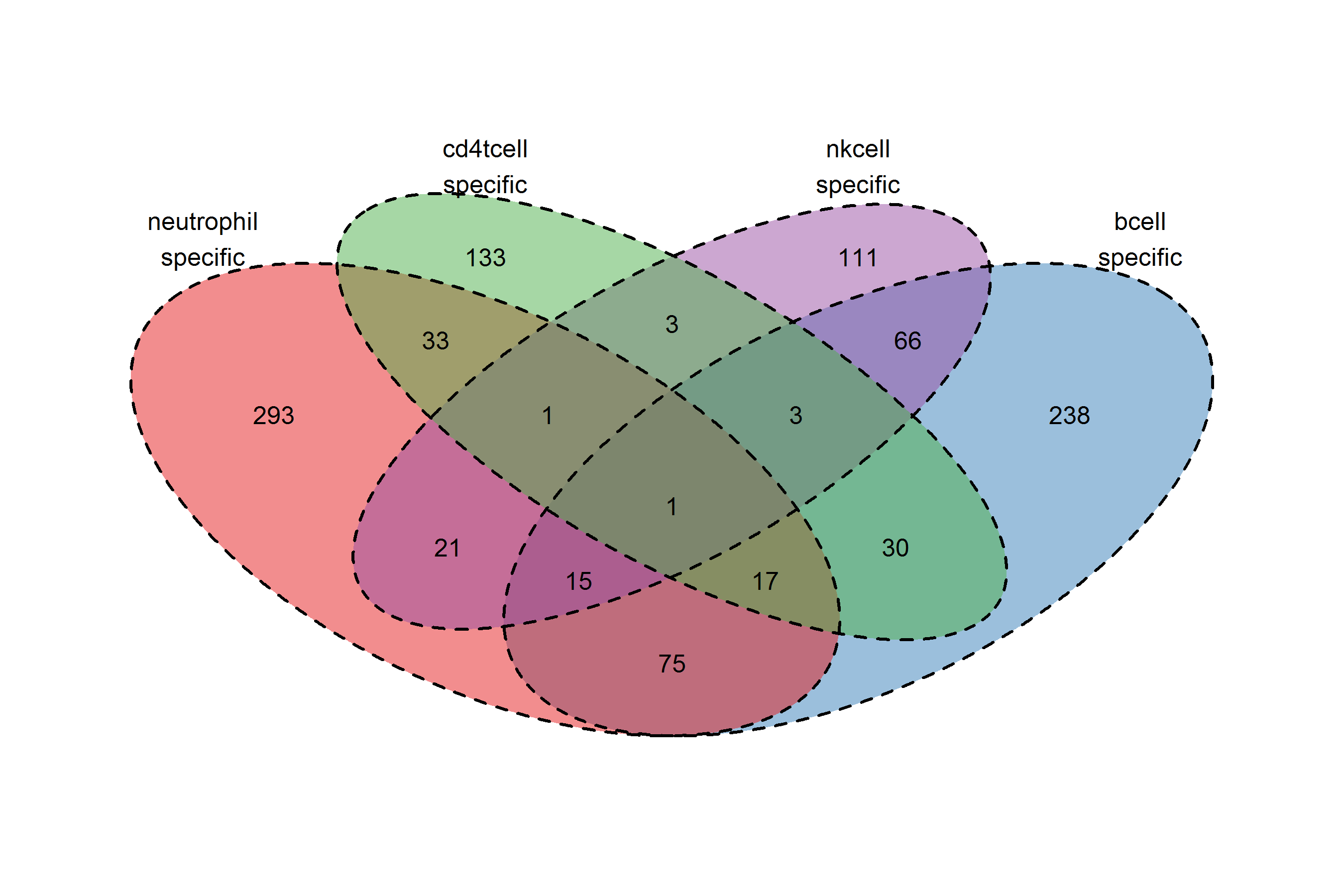

Supplement: Figure S5 — Overlap between the various cell type-specific differentially expressed probe-set lists at the time of rejection. A Venn diagram showing the overlap between the various cell type-specific differentially expressed probe-set lists obtained in Figure 2 . (TIFF) [file pone.0095224.s005.tiff]
